# Impact of bleeding-related complications and/or blood product transfusions on hospital costs in inpatient surgical patients

**DOI:** 10.1186/1472-6963-11-135

**Published:** 2011-05-31

**Authors:** Michael E Stokes, Xin Ye, Manan Shah, Katie Mercaldi, Matthew W Reynolds, Marcia FT Rupnow, Jeffrey Hammond

**Affiliations:** 1 United BioSource Corporation, Montréal, QC, Canada; 2 Ethicon Inc., Somerville, NJ, USA; 3 Xcenda, Palm Harbor, FL, USA; 4 United BioSource Corporation, Lexington, MA, USA

## Abstract

**Background:**

Inadequate surgical hemostasis may lead to transfusion and/or other bleeding-related complications. This study examines the incidence and costs of bleeding-related complications and/or blood product transfusions occurring as a consequence of surgery in various inpatient surgical cohorts.

**Methods:**

A retrospective analysis was conducted using Premier's Perspective™ hospital database. Patients who had an inpatient procedure within a specialty of interest (cardiac, vascular, non-cardiac thoracic, solid organ, general, reproductive organ, knee/hip replacement, or spinal surgery) during 2006-2007 were identified. For each specialty, the rate of bleeding-related complications (including bleeding event, intervention to control for bleeding, and blood product transfusions) was examined, and hospital costs and length of stay (LOS) were compared between surgeries with and without bleeding-related complications. Incremental costs and ratios of average total hospital costs for patients with bleeding-related complications vs. those without complications were estimated using ordinary least squares (OLS) regression, adjusting for demographics, hospital characteristics, and other baseline characteristics. Models using generalized estimating equations (GEE) were also used to measure the impact of bleeding-related complications on costs while accounting for the effects related to the clustering of patients receiving care from the same hospitals.

**Results:**

A total of 103,829 cardiac, 216,199 vascular, 142,562 non-cardiac thoracic, 45,687 solid organ, 362,512 general, 384,132 reproductive organ, 246,815 knee/hip replacement, and 107,187 spinal surgeries were identified. Overall, the rate of bleeding-related complications was 29.9% and ranged from 7.5% to 47.4% for reproductive organ and cardiac, respectively. Overall, incremental LOS associated with bleeding-related complications or transfusions (unadjusted for covariates) was 6.0 days and ranged from 1.3 to 9.6 days for knee/hip replacement and non-cardiac thoracic, respectively. The incremental cost per hospitalization associated with bleeding-related complications and adjusted for covariates was highest for spinal surgery ($17,279) followed by vascular ($15,123), solid organ ($13,210), non-cardiac thoracic ($13,473), cardiac ($10,279), general ($4,354), knee/hip replacement ($3,005), and reproductive organ ($2,805).

**Conclusions:**

This study characterizes the increased hospital LOS and cost associated with bleeding-related complications and/or transfusions occurring as a consequence of surgery, and supports implementation of blood-conservation strategies.

## Background

Bleeding can be a complication of surgery that may lead to substantial morbidity and mortality. In cardiac surgery, severe bleeding occurs in approximately 7% of cases [1] and is associated with an increased risk of post-operative mortality [2]. Mortality rates approaching 20% have been observed in elective vascular patients with severe bleeding complications [3]. In trauma patients, uncontrolled bleeding is the cause of between 30%-40% of all trauma-related deaths [4]. Bleeding complications may also occur from percutaneous procedures that are not considered surgical [5]. Approximately 7% of percutaneous coronary interventions (PCI) performed emergently in patients with acute coronary syndrome result in major bleeding [6]. As with procedures classified as surgical, severe bleeding from PCI has also been linked to significant increases in morbidity and mortality [7].

Blood transfusions, which can be planned/expected or unexpected, and reoperations are used to control bleeding and avert deaths. These procedures are often costly and require large amounts of clinical and staff resources [1]. Blood transfusions are not without considerable risks including nosocomial infection [8,9], immunosuppression [10], transfusion-related acute lung injury [11], and even death [12,13]. These complications may add additional costs to the hospitalization and even post-discharge medical care [1].

The purpose of this study was to estimate the economic burden associated with bleeding-related complications and/or transfusions occurring as a consequence of surgery in eight inpatient surgical cohorts, including cardiac, vascular, non-cardiac thoracic, solid organ, general surgery, knee/hip replacement, reproductive organ, and spinal surgery. These procedures represent the majority of all inpatient surgical procedures performed in the United States (US)[14], and inpatient data on the costs of treating patients with bleeding-related complications in these procedure groups are limited.

## Methods

### Data Source

Data for this retrospective cohort study were obtained from Premier's Perspective™ Comparative Database (PCD)[15]. The current study used PCD data containing inpatient, clinical, drug utilization, and hospital billing data from more than 600 hospitals throughout the US. Premier collects data from participating hospitals in its healthcare alliance. The Premier healthcare alliance was formed for hospitals to share knowledge, improve patient safety, and to reduce risks. Participation in the Premier healthcare alliance is voluntary. The PCD is comprised of hospital administrative data from the United States and, although the PCD excludes federally-funded hospitals (e.g., Veterans Affairs), the hospitals included are nationally representative based on bed size, geographic location, and teaching hospital status [16]. Approximately 5 million new hospital discharges are added to the database each year. According to the Healthcare Utilization Project (HCUP) data, there were 39.5 million hospital discharges throughout the US in 2007 [17]. Therefore, the Premier data used at the time of this analysis represented approximately 13% of all US hospital discharges. All hospitals participating in the healthcare alliance submit data on all patients, payors, and providers as captured on the hospital billing record to Premier. The data go through quality assurance and validation checks and once the data have been validated the information is added to the database. The PCD contains a patient-level date-stamped log for all procedures, medications, laboratory, and diagnostic services rendered during the hospital stay. Data elements include hospital and patient identifiers, primary and secondary ICD-9-CM diagnosis and procedure codes, length of hospital stay, admission type, and primary payer. Also available are data elements for demographic and hospital characteristics, including age, race, geographic location of provider, hospital bed size, teaching hospital status, and hospital location (urban or rural). Hospitals submit demographic data including age and patient reported race (White, Black, Hispanic, American Indian, Asian/Pacific Islander, and Other). Data on hospital characteristics including hospital bed size, teaching hospital status (yes/no), and hospital location (urban/rural) are reported by hospitals. The geographic location of the provider (New England, Middle Atlantic, East North Central, West North Central, South Atlantic, East South Central, West South Central, Mountain, and Pacific) is based on Centers for Medicare and Medicaid Services metropolitan statistical area regions. Patient and provider information contained within the PCD are de-identified making it fully compliant with the Health Insurance Portability and Accountability Act privacy regulations. Institutional review board (IRB) approval for this study was not required as dictated by Title 45 CFR (Code of Federal Regulations) Part 46 of the United States under the exemption that this research involved the study of existing data and that the information was recorded in such a manner that the subjects could not be identified, directly or through identifiers linked to the subjects [18]. Hence, IRB approval was not obtained.

### Patient Population

All patients in the PCD undergoing an inpatient surgery of interest (cardiac, vascular, non-cardiac thoracic, solid organ, general, knee/hip replacement, reproductive organ, and spinal surgery) from January 1, 2006 to December 31, 2007 were selected for inclusion. Patients of all ages were included in this study. Procedures related to surgeries of interest were identified using the International Classification of Diseases, Ninth Revision-Clinical Modification (ICD-9-CM) procedure codes (Table 1). For patients with multiple hospitalizations, the most recent hospitalization (i.e., index) was selected for analysis to maximize the pre-index study period available for an assessment of baseline clinical and demographic characteristics. Patients were excluded if they were transferred from another hospital or an unknown source to ensure that complete hospitalization data were available for every patient. Patients were then classified into study subgroups by procedure type (cardiac, vascular, non-cardiac thoracic, solid organ, general surgery, knee/hip replacement, reproductive organ, and spinal surgery). A minority of patients (approximately 5%) was included in > 1 study subgroup because they were operated on in surgical sites spanning multiple surgical categories during the index hospitalization. For example, patients who had an operation on the vessels of their head and neck in addition to their heart valves would have been included in the separate vascular and cardiac cohort analyses, respectively.

**Table 1:** ICD-9-CM Procedure Codes Used to Identify Surgical Subgroups

Type of Surgery	ICD-9-CM Procedure Codes
Cardiac	
Heart Valves	35.00-35.99
Heart Vessels	36.03, 36.10-36.2, 36.91-36.99
Other Operations on Heart and Pericardium	37.10-37.12, 37.31-37.54, 37.62-37.68
Miscellaneous	39.0, 39.61-39.64, 39.66, 33.6
Vascular	
Incision, Excision, and Occlusion of Vessels	38.00-38.18, 38.30-38.49, 38.60-38.89
Other Operations on Vessels	39.21-39.41, 39.49
Non-cardiac Thoracic	
Lung and Bronchus	32.01-32.21, 32.24, 32.25, 32.28-32.9
Other Operations on Lung and Bronchus	33.0-33.52, 33.91-33.99
Chest Wall, Pleura, Mediastinum, and Diaphragm	34.01-34.21, 34.24-34.27, 34.29-34.59, 34.72-34.89, 34.93-34.99
Esophagus	42.01, 42.09, 42.10-42.12, 42.19, 42.40-42.42, 42.51-42.59, 42.61-42.69, 42.7
General	
Stomach	43.0, 43.19-43.3, 43.42-44.11, 44.15, 44.21, 44.29-44.43, 44.49-44.91, 44.95-44.99
Intestine	45.11, 45.13-45.15, 45.19, 45.26-45.41, 45.49-46.99
Gall Bladder	51.04, 51.13, 51.22-51.83, 51.89-51.94
Solid Organ	
Pancreas	52.01-52.09, 52.12, 52.21-52.96, 52.99
Kidney	55.11, 55.12, 55.24, 55.31-55.91, 55.95-55.99
Liver	50.0, 50.12-50.99
Spleen	41.1, 41.2, 41.41-41.5, 41.93-41.99
Knee/Hip Replacement	
Knee	81.54, 00.80-00.83
Hip	80.05, 81.51, 81.52, 81.53, 00.70-00.73, 00.85-00.87
Reproductive Organ	
Male	60.0-63.71, 63.81-63.99, 64.11, 64.2-64.3, 64.42-64.45, 64.92, 64.93, 64.95-64.99
Female	65.01-66.11, 66.2-69.6, 69.91, 69.93-69.99, 70.12-70.14, 70.23-70.29, 70.32-71.11, 71.3-71.9
Spinal Surgery	03.2×, 03.4×, 03.5×, 03.6×, 84.6×, 84.8×, 81.0×, 81.3×, 81.62-81.64

ICD-9-CM = The International Classification of Diseases, Clinical Modification, Ninth Revision

### Bleeding-related Complication and/or Blood Product Transfusion status

Within each procedure cohort, patients were classified into exposure groups according to whether a bleeding-related complication and/or blood product transfusion occurred during the hospital stay. Bleeding-related complications and/or blood product transfusions were identified if the hospital record contained any of the following codes: ICD-9-CM diagnosis codes for hemorrhages or hematomas complicating a procedure (998.11 and 998.12); ICD-9-CM procedure codes for interventions, including return to the operating room to control for bleeding (34.09, 21.0×, 42.33, 45.43, 44.44, 44.49, 54.19, 39.41, 60.94, 06.02, 28.7, 34.03, 54.12, 49.95, and 57.93); ICD-9-CM diagnosis codes for blood product transfusions (V58.2, E879.8, E873.0, E934.7, and E876.0); or ICD-9-CM procedure codes for blood product transfusions (99.00-99.09).

### Study Outcomes

The outcomes measured in this study included the total length of stay (LOS) in days, the number of days spent in an intensive care unit (ICU), and total hospital costs. Total hospital costs were the actual treatment costs incurred by the hospital and were the sum of the hospital's direct and overhead costs. Direct costs represented services that were used by the patient and included items such as the costs of physicians employed directly by the hospital, treatment costs, and food [19]. Direct costs are recorded in the hospital accounting system as they get itemized for each patient and represent the costs of providing care from the hospital's perspective. Cost variables are not tied to reimbursement or payments from health insurers. Overhead costs were costs associated with the overall functioning of the hospital and included nursing, administrative, and management staffing costs, as well as electricity and the depreciation of medical equipment [19]. These costs are added to each patient record in the hospital accounting system as a standard cost according to the hospital department(s) providing care. Costs accrued during the 2006 study calendar year were standardized to 2007 $US using the medical care component of the Consumer Price Index.

### Data Analyses

The proportion of patients experiencing a particular type of complication was assessed according to surgical subgroup. Baseline demographic, clinical, and hospital characteristics were evaluated according to bleeding-related complication and/or blood product transfusion status for all surgical subgroups combined. Pre-operative use of substances that promote hemostasis was defined as receipt of a hemostat in any of the hemostat classes including oxidized regenerated cellulose, collagen, gelatin, fibrin sealant, thrombin, flowables, combination products, and adhesion prevention. Group comparisons were made between patients with and without complications using 2-sided Pearson chi square and *t*-test statistics for categorical and continuous measures, respectively. Statistical significance was evaluated at alpha = 0.05.

The total cost of hospitalization was analyzed for patients with bleeding-related complications and/or blood product transfusions versus those without a bleeding-related complication or transfusion. Patients with hospital costs exceeding $1,000,000 were excluded from the analysis of costs. Multivariate ordinary least squares regression models were created for each surgical population to measure the impact of bleeding-related complications and/or blood product transfusion events on total hospital costs after controlling for important baseline and clinical patient characteristics. As cost data generally do not follow a normal distribution and are often right-skewed, total costs were transformed to their natural logs. Adjusted mean log costs were then retransformed using Duan's smearing estimate [20]. Baseline parameters, clinical characteristics, and comorbid conditions deemed to have an impact on total hospital costs were considered for inclusion into multivariate models, including bleeding-related complication and/or blood product transfusion status, age, gender, race, geographic region, hospital location, year of surgery, teaching hospital status, hospital bed size, surgical admission type (urgent, elective, or emergency), any cause hospitalization within 6 months prior to the index hospitalization, surgery at multiple sites, and comorbid conditions as independent variables. Comorbid conditions included thrombocytopenia, myocardial infarction (MI), hypertension, non-MI coronary disease, diabetes, peripheral vascular disease, obesity, chronic obstructive pulmonary disease (COPD), renal disease, congestive heart failure, cancer, deep vein thrombosis, and liver cirrhosis. All comorbidities were modeled as separate covariates. Statistical comparisons across groups were conducted using a t-test evaluating the null hypothesis that the parameter estimate for bleeding-related complications and/or blood product transfusions is equal to 0. Statistical significance was evaluated at alpha = 0.05. The incremental difference in adjusted mean hospital costs between patients with a bleeding event and/or blood product transfusion and those without these events was assumed to be the hospital costs attributable to having a bleeding event and/or blood product transfusion. Generalized estimating equation (GEE) models with a Gamma distribution and log link function were also used to measure the impact of bleeding-related complications and/or blood product transfusion events on total hospital costs while accounting for the effects related to the clustering of patients receiving care from the same hospitals. The same baseline parameters used for the ordinary least squares regression models of total hospital costs were included in GEE models.

## Results

Table 2 displays the numbers of patients who developed a bleeding-related complication and/or received a blood product transfusion during the surgical hospitalization. Results are displayed by complication type and surgical subgroup. The percentage of patients with any type of bleeding-related complication varied according to subgroup (general: 27.5%; cardiac: 47.4%; solid organ: 28.5%; non-cardiac thoracic: 34.3%; vascular: 31.5%; knee/hip replacement: 29.8%; reproductive organ: 7.5%; spine: 15.0%; and overall: 29.9%). The most common type of event was blood product transfusion, which occurred in 21.2% of all patients (cardiac: 45.8%; vascular: 29.1%; non-cardiac thoracic: 28.5%; solid organ: 26.8%; general: 25.0%; knee/hip replacement: 29.3%; reproductive organ: 6.4%; and spine: 14.3%). The percentages of patients who had an intervention to control for bleeding ranged from 0.0%-9.7% (cardiac: 3.1%; vascular: 2.0%; non-cardiac thoracic: 9.7%; solid organ: 2.9%; general: 6.4%; knee/hip replacement: 0.0%; reproductive organ: 0.5%; and spine: 0.3%). Overall, interventions for bleeding control were relatively uncommon, occurring in only 2.5% of all surgical patients.

**Table 2:** Percentages of Patients with Specific Complication Events, by Surgical Subgroup

Complication	Cardiac	Vascular	Non-cardiac Thoracic	Solid Organ	General	Reproductive Organ	Knee/Hip Replacement	Spinal
Total number of patients	103,829	216,199	142,562	45,687	362,512	384,132	246,815	107,187
Bleeding event only, %	0.9%	1.9%	0.7%	1.0%	0.5%	0.8%	0.5%	0.5%
Re-operation to control bleeding only, %	0.4%	0.4%	4.7%	0.6%	1.9%	0.2%	0.0%	0.2%
Blood product transfusion only, %	40.9%	25.2%	22.7%	22.8%	19.9%	5.7%	28.6%	13.6%
Bleeding event and blood product transfusion, %	2.5%	2.4%	1.2%	1.8%	0.7%	0.5%	0.7%	0.6%
Bleeding event and re-operation to control bleeding, %	0.3%	0.1%	0.4%	0.1%	0.1%	0.1%	0.0%	0.0%
Re-operation to control bleeding and blood product transfusion, %	1.0%	1.1%	3.3%	1.5%	4.1%	0.1%	0.0%	0.1%
Bleeding event, re-operation to control bleeding and blood product transfusion, %	1.4%	0.4%	1.3%	0.7%	0.3%	0.1%	0.0%	0.0%
Any bleeding-related consequences, %	47.4%	31.5%	34.3%	28.5%	27.5%	7.5%	29.8%	15.0%

Baseline demographic and clinical characteristics are presented in Table 3 according to bleeding-related complication and/or blood product transfusion status. All differences in baseline demographic and clinical measures were highly significant across study groups due to the large sample size. Patients less than 18 years of age were included in this study and represented 2.5%, 1.2%, 4.6%, 3.7%, 2.6%, 0.03%, 0.9%, and 2.2% of patients in the cardiac, vascular, non-cardiac thoracic, solid organ, general, knee-hip replacement, reproductive organ, and spinal surgery subgroups, respectively (data not shown). It was possible for some patients to be operated on in surgical sites spanning multiple surgical categories during the index hospitalization. These patients accounted for 15.5%, 15.1%, 20.2%, 41.8%, 11.3%, 1.0%, 3.2%, and 5.1% of patients in the cardiac, vascular, non-cardiac thoracic, solid organ, general, knee-hip replacement, reproductive organ, and spinal surgery subgroups, respectively (data not shown).

**Table 3:** Baseline Demographics and Clinical Characteristics, by Bleeding-related Complication and/or Blood Product Transfusion Status

Characteristic	Complication*	No Complication	*P*-value
Number of patients	351,065	1,175,208	
Age, mean years (SD)	64.0 (17.9)	55.1 (18.7)	< 0.001
Range	1-89	1-89	
Male, N (%)	159,893 (45.5%)	432,211 (36.8%)	< 0.001
Race, N (%)			
White	231,146 (65.8%)	770,920 (65.6%)	< 0.001
Black	46,288 (13.2%)	143,269 (12.2%)	
Hispanic	12,842 (3.7%)	58,358 (5.0%)	
Other	60,789 (17.3%)	202,661 (17.2%)	
Geographic Region, N (%)			
Northeast	57,899 (16.5%)	163,160 (13.9%)	< 0.001
South	170,133 (48.5%)	537,047 (45.7%)	
Midwest	60,598 (17.3%)	259,391 (22.1%)	
West	58,970 (16.8%)	204,439 (17.4%)	
Unknown	3,465 (1.0%)	11,171 (1.0%)	
Primary Payer, N (%)			
Medicare	202,495 (57.7%)	443,685 (37.8%)	< 0.001
Medicaid	24,477 (7.0%)	110,654 (9.4%)	
Private	98,132 (28.0%)	524,710 (44.6%)	
Uninsured	13,495 (3.8%)	45,219 (3.8%)	
Other	12,466 (3.6%)	50,940 (4.3%)	
Comorbidity, N (%)			
Diabetes	162,097 (46.2%)	306,512 (26.1%)	< 0.001
Obesity	41,646 (11.9%)	150,510 (12.8%)	< 0.001
COPD	88,506 (25.2%)	219,635 (18.7%)	< 0.001
Cancer	80,506 (22.9%)	198,206 (16.9%)	< 0.001
Cirrhosis	11,034 (3.1%)	9,835 (0.8%)	< 0.001
Prior Hospitalization, N (%)	161,956 (46.1%)	486,348 (41.4%)	< 0.001
Prior Surgery, N (%)	106,064 (30.2%)	293,533 (25.0%)	< 0.001
Pre-operative use of substances that promote hemostasis, N (%)	7,957 (2.3%)	21,338 (1.8%)	< 0.001

N = number of patients, SD = standard deviation

*Bleeding complication and/or blood product transfusion

The authors interpreted differences between groups to be important for age, gender, primary payer, having a prior surgery as well as comorbidities including diabetes, COPD, and cancer. Patients who had a bleeding-related complication were on average 8.9 years older compared to patients without a complication (mean age 64.0 years vs. 55.1, *P*< 0.001). There were also relevant differences across study groups with respect to the patients' primary payer of medical care. There was a higher percentage of patients experiencing a bleeding-related complication and/or blood product transfusion who received Medicare reimbursement versus patients without these events (57.7% vs. 37.8%, *P*< 0.001). A higher percentage of male patients had a bleeding-related complication versus those without a complication (45.5% vs. 36.8%, *P <*0.001). Patients with bleeding-related complications and/or blood product transfusions were more likely to have diabetes (46.2% vs. 26.1%, *P*< 0.001), COPD (25.2% vs. 18.7%, *P <*0.001), and cancer (22.9% vs. 16.9%, *P <*0.001) compared to those without these events. We interpreted statistically significant differences with respect to demographic characteristics such as race and geographic region as unimportant. Additionally, differences in prior hospitalization, pre-operative use of substances promoting hemostasis, liver cirrhosis, and obesity were deemed unimportant.

Table 4 reports the characteristics of the hospital in which the surgery occurred. There were no relevant differences in hospital size, hospital location (urban vs. rural), or the proportion of patients treated in a teaching hospital across study groups. However, differences were statistically significant due to large sample sizes. The authors interpreted the differences between study subgroups to be unimportant.

**Table 4:** Hospital Characteristics, by Bleeding-related Complication and/or Blood Product Transfusion Status

Characteristic	Complication*	No Complication	*P*-value
Number of patients	351,065	1,175,208	
Hospital size--beds, N (%)			
50-249	61,300 (17.5%)	219,235 (18.7%)	< 0.001
250-749	250,774 (71.4%)	829,201 (70.6%)	
750+	38,991 (11.1%)	126,772 (10.8%)	
Teaching hospital, N (%)	153,739 (43.8%)	505,674 (43.0%)	< 0.001
Hospital location, N (%)			
Urban	313,037 (89.2%)	1,043,060 (88.8%)	< 0.001
Rural	34,563 (9.8%)	120,977 (10.3%)	
Unknown	3,465 (1.0%)	11,171 (1.0%)	
Hospital length of stay			
Mean (SD)	10.4 (13.5)	4.5 (5.4)	< 0.001
Median (Range)	6.0 (1-615)	3 (1-495)	

N = number of patients, SD = standard deviation

*Bleeding complication and/or blood product transfusion

**Figure 1:**
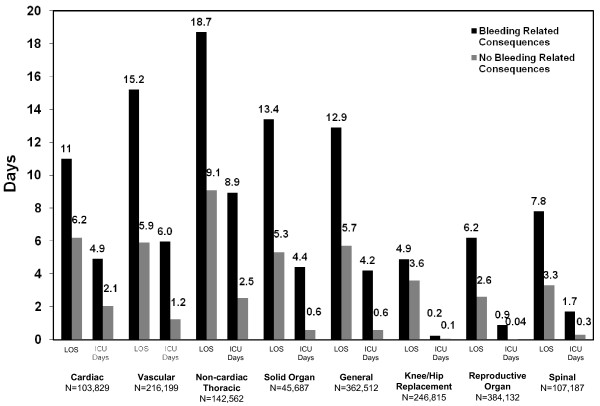
**Mean Hospital LOS and ICU Days by Surgical Cohort and Complication Status**.

Data on hospitalization lengths of stay (data presented in days and unadjusted for covariates) are presented in Figure 1 by surgical cohort and complication status. Patients with bleeding-related complications and/or blood product transfusions experienced a longer LOS compared to patients without bleeding-related complications and blood product transfusions (overall: 10.4 vs. 4.4 days; cardiac: 11.0 vs. 6.2 days; vascular: 15.2 vs. 5.9 days; solid organ: 13.4 vs. 5.3 days; non-cardiac thoracic: 18.7 vs. 9.1 days; general: 12.9 vs. 5.7 days; knee/hip replacement: 4.9 vs. 3.6 days; reproductive organ: 6.2 vs. 2.6 days; and spine: 7.8 vs. 3.3 days). Patients with bleeding-related complications and/or blood product transfusions in all surgical categories also spent more days in an ICU versus patients without bleeding-related complications and/or blood product transfusions (overall: 3.3 vs. 0.5 days; cardiac: 4.9 vs. 2.1 days; vascular: 6.0 vs. 1.2 days; solid organ: 4.4 vs. 0.6 days; non-cardiac thoracic: 8.9 vs. 2.5 days; general: 4.2 vs. 0.6 days; knee/hip replacement: 0.2 vs. 0.1 days; reproductive organ: 0.9 vs. 0.04 days; and spine: 1.7 vs. 0.3 days). Lengths of stay differences were unadjusted for covariates.

Table 5 reports unadjusted total hospitalization costs in 2007 US constant dollars. Total costs were higher among patients with a bleeding-related complication and/or blood transfusion versus those without a complication (cardiac: $43,125 vs. $26,073; vascular: $40,116 vs. $14,288; non-cardiac thoracic: $50,081 vs. $19,213; solid organ: $41,667 vs. $16,480; general: $28,499 vs. $11,937; knee/hip replacement: $18,973 vs. $14,966; reproductive organ: $13,943 vs. $6,220; and spine: $41,917 vs. $20,511).

**Table 5:** Unadjusted Total Inpatient Costs (2007 $US), by Surgical Category and Bleeding-related Complication and/or Blood Product Transfusion Status

Study Measure	Complication*	No Complication
Cardiac		
Number of Patients	49,293	54,533
Mean (SD)	$43,125 ($38,283)	$26,073 ($22,083)
Median (Range)	$33,468 ($24,921-$47,466)	$22,512 ($14,620-$31,365)
Vascular		
Number of Patients	68,211	147,980
Mean (SD)	$40,116 ($49,461)	$14,288 ($19,146)
Median (Range)	$24,989 ($13,664-$46,806)	$9,020 ($5,666-$15,952)
Non-cardiac thoracic		
Number of Patients	48,854	93,699
Mean (SD)	$50,081 ($57,926)	$19,213 ($25,388)
Median (Range)	$32,695 ($16,749-$62,442)	$12,334 ($7,387-$21,127)
Solid organ		
Number of Patients	12,988	32,699
Mean (SD)	$41,667 ($49,699)	$16,480 ($20,877)
Median (Range)	$24,390 ($14,772-$47,692)	$11,044 ($7,890-$17,125)
General		
Number of Patients	99,812	262,695
Mean (SD)	$28,499 ($42,485)	$11,937 ($14,291)
Median (Range)	$15,493 ($7,980-$31,092)	$8,840 ($5,934-$13,325)
Knee/hip replacement		
Number of Patients	73,795	173,020
Mean (SD)	$18,973 ($12,247)	$14,966 ($7,075)
Median (Range)	$16,256 ($13,094-$21,246)	$13,742 ($11,221-$17,209)
Reproductive organ		
Number of Patients	28,951	355,180
Mean (SD)	$13,943 ($19,905)	$6,220 ($5,009)
Median (Range)	$8,849 ($6,078-$14,413)	$5,262 ($4,024-$7,090)
Spinal		
Number of Patients	16,068	91,117
Mean (SD)	$41,917 ($34,525)	$20,511 ($16,114)
Median (Range)	$32,813 ($22,393-$49,884)	$16,557 ($10,541-$25,709)

SD = standard deviation

*Bleeding complication and/or blood product transfusion

Note: 15 patients were excluded from the economic analysis because their hospital costs exceeded $1,000,000

**Figure 2:**
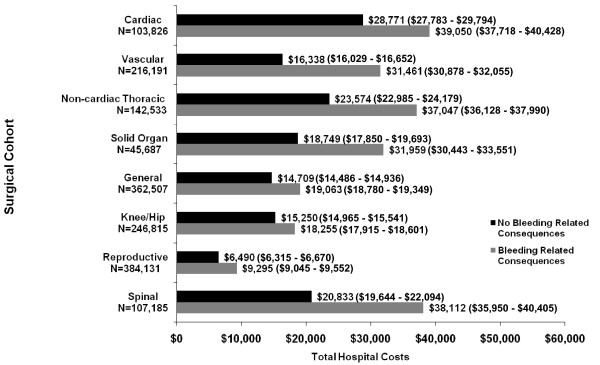
**Mean Total Adjusted^1 ^Hospital Costs (95% CIs) in 2007 $US by Surgical Cohort**.

Figure 2 presents mean total hospital costs by surgical subgroup and complication status. Results were adjusted for differences in baseline clinical, demographic, and hospital characteristics between the bleeding-related complication and/or blood product transfusion and no bleeding-related complication and/or blood product transfusion study groups using multivariate ordinary least squares regression modelling techniques. In all surgical subpopulations, the bleeding-related complication and/or blood product transfusion groups had higher total hospital costs versus those without a bleeding-related complication and/or blood product transfusion (*P*< 0.0001). There were large variations in the incremental differences in mean total costs (bleeding-related complication-no complication) for each of the surgical subgroups (cardiac: $10,279; vascular: $15,123; non-cardiac thoracic: $13,473; solid organ: $13,210; general: $4,354; knee/hip replacement: $3,005; reproductive organ: $2,805; and spine: $17,279).

Table 6 reports the ratio of average total costs for patients with bleeding-related complications versus those without bleeding-related complications, adjusted for baseline, clinical characteristics and the effects related to the clustering of patients receiving care from the same hospitals using GEE models. Ordinary least squares model results are also presented for comparison. The ratio of average total costs estimated using GEE models for patients with bleeding complications vs. those without complications ranged from 1.31 to 1.93 for cardiac and vascular surgery, respectively. All average total cost ratios were statistically significant (*P <*0.001). Cost ratios were similar to those observed using OLS regression with the exception of the ratio for general surgery (1.46 vs. 1.34 for GEE and OLS models, respectively).

**Table 6:** Impact of Bleeding Complication Status on Total Hospital Costs: Comparison of GEE and OLS Models

Surgical Cohort	GEE		OLS	
	Model Estimate (CI)*	***P*-value**	Model Estimate (CI)*	***P*-value**

Cardiac	1.31 (1.30, 1.32)	< 0.001	1.36 (1.35, 1.36)	< 0.001
Vascular	1.93 (1.93, 1.95)	< 0.001	1.93 (1.92, 1.95)	< 0.001
Non-cardiac thoracic	1.60 (1.58, 1.60)	< 0.001	1.57 (1.55, 1.58)	< 0.001
Solid organ	1.72 (1.68, 1.73)	< 0.001	1.70 (1.68, 1.72)	< 0.001
General	1.46 (1.46, 1.48)	< 0.001	1.34 (1.34, 1.35)	< 0.001
Knee/hip	1.22 (1.21, 1.22)	< 0.001	1.20 (1.20, 1.21)	< 0.001
Reproductive organ	1.49 (1.48, 1.51)	< 0.001	1.43 (1.42, 1.43)	< 0.001
Spinal	1.79 (1.77, 1.80)	< 0.001	1.82 (1.80, 1.84)	< 0.001

CI = confidence interval, GEE = generalized estimating equations, OLS = ordinary least squares

*Ratio of average total costs for patients with bleeding-related complications versus those without complications

Note: 15 patients were excluded from the economic analysis because their hospital costs exceeded $1,000,000.

GEE model estimated by gamma log link function with total costs as the dependent variable, adjusted for baseline clinical, demographic, hospital characteristics and clustering on hospital.

OLS model with log total costs as the dependent variable, adjusted for baseline clinical, demographic, and hospital characteristics.

## Discussion

We estimated the hospital costs associated with bleeding-related complications in 8 surgical cohorts, including cardiac, vascular, non-cardiac thoracic, solid organ, general surgery, knee/hip replacement, reproductive organ, and spinal surgery, using patient-level data from Premier's PCD. Patients undergoing the surgical procedures of interest during calendar years 2006 and 2007 were identified using ICD-9-CM procedure codes. For each surgical cohort, patients with bleeding-related complications and/or blood product transfusions and those without these events were further delineated using a combination of diagnosis and procedure codes. Mean hospital costs were calculated for each study group after adjusting for differences in baseline demographic and clinical characteristics deemed to have an impact on costs. The incremental difference in adjusted mean costs between patients with a bleeding event and/or blood product transfusion and those without these events was assumed to be an estimate of the hospital costs attributable to having a bleeding event and/or blood product transfusion.

Results of our analyses indicate that bleeding events and/or blood product transfusions were relatively common during the surgical hospitalization and varied according to surgical cohort (general: 27.5%; cardiac: 47.4%; solid organ: 28.5%; non-cardiac thoracic: 34.3%; vascular: 31.5%; knee/hip replacement: 29.8%; reproductive organ: 7.5%; and spinal surgery: 15.0%). Among the types of events identified in this study, blood product transfusions were the most common, occurring in approximately 21.2% of the patient cohort. Interventions for bleeding control, in contrast, were less common and occurred in only 2.5% of patients.

In analyses of costs adjusted for disparities in baseline and clinical characteristics, we observed large variations in the incremental difference in mean total costs between patients with a bleeding-related complication and/or blood product transfusion and those without these events for each surgical cohort. Patients with bleeding-related complications and/or blood product transfusions experienced longer hospital lengths of stay and spent more time in the ICU compared to patients without these events.

Studies investigating costs related to bleeding-related complications reveal that they vary widely depending on the surgical cohort. For example, hospital costs attributable to bleeding in trauma patients are reported to be much higher ($38,628) compared to patients undergoing more routine procedures such as PCI ($5,883) and knee and hip surgery ($7,593) [21-23]. Our inpatient cost estimates attributable to bleeding-related complications and/or blood product transfusions ranged from $2,805-$17,279 for reproductive and spinal surgery, respectively. Considering that the surgical procedures examined in this study were less complex compared to trauma surgery, it makes sense that our estimates are lower than what is previously reported for trauma patients.

This study is subject to several limitations. First, the identification of patients who had a bleeding event and/or blood product transfusion was based on an algorithm utilizing a combination of ICD-9-CM procedure and diagnosis codes as well as billing charges appearing on the hospital record. These coding systems are primarily used for administrative purposes in obtaining reimbursement for the services provided by the hospital. Therefore, we chose to incorporate both bleeding events as well as blood product transfusions in our definition of the study groups. This distinction is important because, with certain surgical procedures, blood product transfusions are routinely administered as part of the surgery and not necessarily because the patient had unexpected bleeding requiring transfusion. Thus, we felt we could not have reliably distinguished one transfusion type from the other. Additionally, it was not possible to differentiate between transfusions occurring as a consequence of the surgery from transfusions that were the result of an underlying or presenting condition such as vascular trauma or ruptured aortic aneurysms if the transfusion occurred on the same day of the surgery as only data pertaining to the day of the transfusion were available. Therefore, it is possible that some patients within the bleeding-event and/or blood product transfusion group had a transfusion that did not occur as a consequence of the surgery and were misclassified. We did not exclude patients with conditions such as vascular trauma or ruptured aortic aneurysms from this analysis in order to facilitate bleeding occurring as a consequence of the surgery. Patients presenting with aortic aneurysms probably would have undergone either open aortic resection with replacement (38.44) or endovascular repair (39.71). The proportion of vascular surgery patients undergoing open aortic resection with replacement was only 1.8% and patients undergoing endovascular repair were not selected for this analysis. Therefore, we do not expect the inclusion of patients with ruptured aortic aneurysm and the possible misclassification of the bleeding outcome to change results significantly. With respect to vascular trauma, the inclusion of trauma cases that likely had a transfusion as a result of their presenting condition and not the surgical procedure probably introduced some misclassification bias into this study. Since trauma patients have much higher costs relative to patients without trauma, the inclusion of these cases would have had the effect of inflating our bleeding-related complication cost estimates [21]. We suspect that this upward bias is probably minimized by the fact that patients presenting with trauma probably represented only a small percentage of the total patients included in this study. A study from the University of Michigan comparing outcomes between trauma and general surgery patients reported enrolling only 525 patients admitted to the Trauma service in comparison to 54, 478 general surgery patients during 2004 [21].

Second, patients were excluded from analyses if they were transferred from another hospital or an unknown source to ensure that complete hospitalization data were available for every patient. We did not feel that we could reliably identify and link multiple hospitalizations for patients transferred to another hospital using the administration codes in the database. As these transferred patients probably had higher costs relative to the current sample of patients, the exclusion of these patients likely had the effect of underestimating the total inpatient episode of care. Furthermore, if more patients with a bleeding-related complication were transferred to other hospitals versus those without bleeding-related complications, the incremental difference in costs between these groups may in fact be larger than what is currently reported.

Third, pediatric patients < 18 years of age were included in analyses. Although age was included as a covariate in multivariate cost models, one could argue that, because pediatric patients likely underwent different procedures compared to adult patients, separate analyses of these distinct patient groups is warranted. Thus, multivariate cost models examining adult and pediatric patients separately were constructed to provide insight regarding the differences in costs between these two distinct subpopulations. Among adults, patients with bleeding-related complications had higher costs relative to patients without complications (cardiac: $38,686 vs. $28,914, vascular: $30,640 vs. $16,027, non-cardiac thoracic: $36,150 vs. $23,494, solid organ: $31,807 vs. $18,878, general surgery: $18,880 vs. $14,427, knee/hip replacement: $18,248 vs. $15,247, reproductive organ: $9,269 vs. $6,493, and spinal: $37,978 vs. $20,847); results were similar to main analyses and statistically significant. Among the subset of pediatric patients, those with bleeding-related complications had higher costs relative to patients without complications (cardiac: $58,239 vs. $29,514, vascular: $113,822 vs. $39,506, non-cardiac thoracic: $79,898 vs. $35,680, solid organ: $61,122 vs. $28,742, general surgery: $104,505 vs. $37,316, reproductive organ: $34,703 vs. $16,999, and spinal: $54,369 vs. $31,984). A multivariate model was not created for pediatric knee/hip surgery patients because of the small sample size relative to the number of parameters in the model. Overall, costs in patients < 18 years of age were higher compared to the adult population. The incremental difference in costs between patients with bleeding-related consequences and those without bleeding-related consequences was also higher in pediatric patients compared to adults.

Fourth, our analyses of costs were adjusted for differences in baseline clinical and demographic characteristics using multivariate ordinary least squares regression with Duan's smearing back retransformation. The approach used for retransformation should be dependent upon the nature of the error term on the transformed scale [24]. Since the distribution of the error term is usually unknown, reliance on the assumption of normality or homoskedasticity can lead to inconsistent estimates [24]. Therefore, we also examined the sensitivity of results to retransformation using subgroup-specific smearing factors as proposed by Manning [25]. Using this alternate retransformation method, cost estimates for patients with bleeding-related complications remained statistically significantly higher versus those without complications. In fact, the incremental differences in mean total costs (bleeding-related complication - no complication) for each of the surgical subgroups were higher compared to the main study results (cardiac: $17,055, vascular: $25,795, non-cardiac thoracic: $30,963, solid organ: $25,153, general: $16,567, knee/hip replacement: $4,009, reproductive organ: $7,707, and spinal surgery: $21,326).

It should be noted that a small percentage of patients (approximately 5%) were included in more than one study subgroup because they were operated on in surgical sites spanning multiple surgical categories during the index hospitalization. A sensitivity analysis excluding these patients was conducted to examine the effect they might have had on cost results. Results show that their effect was minimal as the incremental difference in mean total costs (bleeding-related complication - no complication) were similar to the main analyses for each of the surgical subgroups (cardiac: $10,391, vascular: $14,639, non-cardiac thoracic: $12,366, solid organ: $12,434, general: $3,974, knee/hip replacement: $3,148, reproductive organ: $3,266, and spinal surgery: $17,448).

All baseline and clinical characteristics deemed to have an impact on total hospital costs were included in our models. Nevertheless, we could not control for certain covariates known to affect costs because this information was not available in the PCD. These covariates included sociodemographic parameters such as smoking status and whether the patient was living alone. Although we identified patients who were obese using ICD-9-CM diagnosis codes (278.00-278.02), to the best of our knowledge it is unknown to what extent these codes can be used to accurately identify individuals who are obese. Additionally, data on certain chronic comorbid conditions were likely underestimated. Comorbidity data were only available if a patient was treated for the condition during the index hospitalization or if a patient was treated in a prior admission to a hospital within Premier's data capture network. Because data on outpatient physician visits are not included in the PCD, for some comorbidities including thrombocytopenia, MI, hypertension, non-MI coronary disease, renal disease, congestive heart failure, and deep vein thrombosis it was impossible to determine whether the condition was chronic and unrelated to the index surgical hospitalization or occurred as an adverse event related to the surgery or bleeding complication. Many patients also did not have prior hospitalization data, therefore many comorbidities were identified within the index surgical hospitalization. As a result, the assumption of independence between covariates in our multivariate analyses may have been violated if, for example, certain conditions such as MI or renal failure developed as a result of the bleeding complication and were not actually pre-existing conditions. As a result regression models could have produced inaccurate estimates of regression coefficients, variability, and *P*-values. Therefore, multivariate cost analyses were also run using only the conditions that were deemed to be obvious chronic conditions identifiable in the database. These conditions included diabetes, obesity, COPD, cancer, and cirrhosis. The incremental cost per hospitalization associated with bleeding-related complications was similar to the main study results ($15,931 for vascular, $15,410 for solid organ, $14,653 for non-cardiac thoracic, $11,715 for cardiac, $5,114 for general, $3,119 for knee/hip replacement, $2,961 for reproductive organ, and $17,707 for spinal surgery) and the difference in costs among patients with bleeding-related complications and those without complications was statistically significant for every surgical cohort (*P <*0.001). We also did not control for conditions such as coagulopathy, anemia, or the use of bone marrow suppressants. If a higher percentage of patients in the bleeding complication group had these prior conditions or procedures, our cost estimates related to bleeding complications may have been overstated especially if a prior hospitalization for these conditions or procedures was associated with higher costs during the study index surgical hospitalization. Finally, information relating to the severity of the comorbid condition was not available. As severity could have an impact on hospital costs, the inability to control for severity is another limitation of our analysis.

## Conclusions

These analyses offer insight into the magnitude of differences in costs between patients with and without bleeding-related complications and/or blood product transfusions among hospital-based procedures in 7 specialties. The optimal management of bleeding is an important goal, as these complications are associated with increased morbidity and mortality in a variety of different surgical populations [2-4,7]. Given the high costs associated with managing patients with bleeding-related complications and/or blood product transfusions, additional comprehensive approaches should be evaluated or developed to optimize intra-operative bleeding management, including product and technique strategies.

## Key Messages

• In every surgical cohort examined, patients with bleeding-related complications and/or blood product transfusions had longer lengths of stays and higher total hospital costs.

• There were large variations in the incremental differences in total hospital costs between bleeding-related complication and no complication cohorts for each of the surgical subgroups (cardiac: $10,279; vascular: $15,123; non-cardiac thoracic: $13,473; solid organ: $13,210; general: $4,354; knee/hip replacement: $3,005; reproductive organ: $2,805; and spine: $17,279).

• Patients with bleeding-related complications and/or blood product transfusions spent, on average, approximately 2.7 more days in an ICU compared to patients without bleeding-related complications and blood product transfusions (overall: 3.3 vs. 0.5 days).

• Given the high costs associated with managing patients with bleeding-related complications and/or blood product transfusions, additional comprehensive approaches should be evaluated or developed to optimize intra-operative bleeding management, including product and technique strategies.

## List of Abbreviations

COPD: Chronic obstructive pulmonary disease; ICU: Intensive care unit; LOS: Length of stay; MI: Myocardial infarction; PCD: Premier's Perspective™ Comparative Database; PCI: Percutaneous coronary intervention; US: United States.

## Competing interests

Michael E. Stokes, Katie Mercaldi, and Matthew W. Reynolds are full-time employees of United BioSource Corporation. United BioSource Corporation received funding from Ethicon, Inc. for the conduct of this study and drafting of the manuscript. Manan Shah is an employee of Excenda, who received funding from Ethicon, Inc. for study consultation. Xin Ye, Marcia FT Rupnow, and Jeffrey Hammond are full-time employees of Ethicon, Inc. and receive a salary from Ethicon.

United BioSource Corporation is a global scientific and medical affairs organization that partners with life science companies to help generate real-world evidence of product effectiveness, safety, and value to assist health care decisions and enhance patient care. Ethicon Inc. is a global medical device company with major products covering wound closure; hernia repair; biosurgery; women's health, aesthetic medicine and ENT. It develops, manufactures and markets a variety of products designed to achieve adjunctive hemostasis. Excenda is a full-service consultancy and managed markets agency that helps manufacturers identify, demonstrate, and deliver their brand's value proposition to healthcare stakeholders. UBC and Excenda are independent consulting firms that were contacted by Ethicon to perform this study.

## Authors' contributions

MES participated in the design and coordination of the study, analysis and interpretation of the data, and the drafting of the manuscript. KM created the study analytic file from the raw data, carried out the statistical analysis, and participated in the revision of the manuscript. XY, MFTR, and JH participated in the design of the study, analysis and interpretation of the data, and revision of the manuscript. MS and MR participated in the design of the study and analysis and interpretation of the data. MFTR and MR were also involved in study conception. All authors read and approved the final manuscript.

## Funding

Funding for this study was provided by Ethicon, Inc.

## Pre-publication history

The pre-publication history for this paper can be accessed here:

http://www.biomedcentral.com/1472-6963/11/135/prepub
